# Generative artificial intelligence literacy profiles and workforce readiness among pre-professional nursing students: a latent profile analysis

**DOI:** 10.3389/fpubh.2026.1851193

**Published:** 2026-06-01

**Authors:** Yanfang Zhang, Xiaomei Ji, Long Zhao, Xiumu Yang

**Affiliations:** School of Nursing, Bengbu Medical University, Bengbu, China

**Keywords:** generative artificial intelligence literacy, latent profile analysis, pre-professional nursing students, technology acceptance model (TAM), workforce readiness

## Abstract

**Background:**

Generative Artificial Intelligence (GenAI) Literacy hold significant implications for pre-professional nursing students’ academic and professional development, potentially influencing their workforce readiness. However, existing studies have overlooked the inter-individual heterogeneity among pre-professional nursing students, and few have explored the correlation between their GenAI Literacy and workforce readiness.

**Purpose:**

This study aims to identify the latent profiles and influencing factors of their GenAI literacy, and to compare the differences in workforce readiness across these latent profiles.

**Methods:**

A cross-sectional design was employed. From November 2025 to February 2026, 750 pre-professional nursing students from different geographical regions in China were recruited. Participants completed the College Students’ GenAI Literacy Scale and the Nursing Practice Readiness Scale. Latent Profile Analysis (LPA) was used to identify the profiles of GenAI Literacy among pre-professional nursing students. Two-category logistic regression was conducted to evaluate the predictors of different profiles. One-way ANOVA and rank-sum test were used to compare the workforce readiness across these profiles.

**Results:**

Three latent profiles of GenAI literacy were identified: Low GenAI literacy group (*n* = 201, 28.2%), Moderate GenAI literacy group (*n* = 399, 56.0%) and the High GenAI literacy group (*n* = 112, 15.8%). Multinomial logistic regression showed that academic performance, receipt of GenAI-related training, understanding of basic GenAI algorithms, GenAI tool usage frequency, and the use of GenAI software in clinical practice/learning were predictive factors of the latent profiles of GenAI literacy. Significant differences were found in the workforce readiness (*p* < 0.05).

**Conclusion:**

Pre-professional nursing students’ GenAI literacy can be classified into three latent profiles. Enhancing GenAI literacy may improve their overall workforce readiness.

## 1 Introduction

Generative Artificial Intelligence (GenAI) signifies a substantial paradigm shift in AI, enabling the autonomous creation of novel and coherent content beyond traditional discriminative tasks (1). Recent advancements have seen models like ChatGPT, DeepSeek, Gemini, Claude, and Llama 3 increasingly applied across various sectors, including education (2), healthcare (3), content creation (4), and immersive digital environments (5). This rapid integration of GenAI is fundamentally reshaping global job markets and workforce competencies, particularly in high-stakes fields such as nursing (6). Employers now seek professionals who can effectively leverage AI tools to solve practical problems and adapt to intelligent work environments, moving beyond traditional professional knowledge and operational skills (7). In the healthcare sector, the accelerating integration of AI technology is imposing new demands on nursing professionals (8). While traditional nursing roles centered on fundamental skills like clinical operations and patient observation, contemporary nursing requires both core clinical competencies and advanced AI literacy (9). This includes the ability to effectively utilize intelligent clinical decision support systems, smart nursing assessment tools, and virtual simulation training to enhance efficiency, ensure quality, and navigate complex clinical scenarios (10).

Pre-professional nursing students are pivotal in shaping the future application of GenAI in nursing (11). Integrating GenAI into nursing education and practice is profoundly transforming their learning pathways, clinical reasoning, and professional capabilities (12). Proficiency with GenAI tools can significantly improve nursing students’ clinical performance and efficiency, cultivate critical thinking and ethical decision-making abilities, bolster professional self-efficacy, and reduce anxiety (13). It also fosters their participation in interdisciplinary collaboration and innovative practices within healthcare (14).

GenAI literacy involves more than mere tool proficiency; it encompasses understanding underlying mechanisms, appreciating applicative potential across diverse contexts, and critically reflecting on ethical implications (15, 16). This distinguishes it from traditional AI literacy through dimensions such as Awareness, Usage, Evaluation, and Ethics and Risks (17). Current literature primarily addresses GenAI literacy within the educational sector, particularly higher education, examining its adoption, interaction, evaluation, and ethical perceptions among both educators and learners (18). Studies reveal varying levels of GenAI literacy among students concerning utilization, interaction, output evaluation, and ethical awareness (19). While nationality and prior AI learning experiences significantly influence GenAI literacy, age and educational attainment do not show significant effects (20). Importantly, GenAI literacy has been recognized as a pivotal competency that positively shapes students’ employability and career competitiveness, enabling them to better adapt to intelligent healthcare environments and meet evolving workforce demands (21). However, large-scale empirical investigations specifically targeting the GenAI literacy of pre-professional nursing students are currently scarce. Future research should focus on developing discipline-specific conceptual frameworks, assessment instruments, and effective interventions to ensure that pre-professional nursing students can responsibly and effectively leverage GenAI technologies to meet the evolving demands and challenges of modern healthcare practice.

Workforce readiness is a multifaceted concept defining the extent to which individuals, particularly recent graduates or new entrants, possess the necessary knowledge, skills, abilities, attitudes, and behaviors to effectively fulfill professional responsibilities in the labor market (22). In the context of nursing, this construct is critical for evaluating whether nursing students or newly graduated nurses can meet the fundamental requirements of clinical positions (23). It encompasses multiple dimensions, serving as a vital bridge between nursing education and clinical practice (23). It reflects the ability of new nurses to adapt to the clinical environment and perform assigned competently during the transition from student to professional (24). Relevant studies have shown that the overall practice readiness of pre-professional nursing students is at a moderate level (25, 26), influenced by personality characteristics, satisfaction with work-life balance, and the clinical learning environment (27).

The Technology Acceptance Model (TAM) is a widely used framework for understanding users’ acceptance of new technologies (28). TAM not only explains individuals’ willingness to accept AI technology but is also linked to their practice readiness. In terms of awareness and perceived usefulness, nursing students with higher AI literacy can more effectively identify the practical value of GenAI in nursing practice, such as its potential to assist clinical decision-making, reduce documentation burden, and accelerate knowledge retrieval (29). In terms of usage ability and perceived ease of use, pre-professional nursing students with strong GenAI application skills—especially proficiency in prompt engineering—can significantly improve their perceived ease of use of GenAI (11). In terms of evaluation ability and perceived usefulness, GenAI evaluation competence enables interns to rationally recognize the value of the technology while identifying limitations and risks in outputs, thereby enhancing trust and judgments of usefulness in clinical applications (30). In terms of ethics and risks and acceptance intention, a high level of AI ethical literacy is essential for nursing students to responsibly adopt and apply GenAI (31). A profound understanding of ethical risks and a responsible attitude can promote the active integration of GenAI into their future clinical practice while avoiding harm caused by inappropriate use (32). However, studies exploring the association between GenAI literacy and workforce readiness among nursing students remain limited at this stage.

Latent Profile Analysis (LPA) is an individual-centered approach used to identify distinct subgroups and compare differences in key indicators across categories. It facilitates the detection of high-risk groups and provides a basis for developing targeted and precise interventions (33). To gain an in-depth understanding of GenAI literacy among pre-professional nursing students, the core objective of this study was to identify heterogeneous profiles of GenAI literacy using LPA, thereby assisting nursing managers and educators in designing tailored training programs. This study aims to identify subgroups of GenAI literacy among pre-professional nursing students using Latent Profile Analysis, and to examine the relationships between these subgroups and workforce readiness, as well as associated influencing factors. The findings will enrich the research system of nursing education and provide evidence for fostering GenAI literacy and improving clinical teaching among pre-professional nursing students.

## 2 Materials and methods

### 2.1 Study participants and procedures

Convenience sampling was adopted to select pre-professional nursing students from 12 Grade A tertiary general hospitals in five provinces/municipalities of China, including Anhui, Jiangsu, Zhejiang, Guangdong, and Shanghai, from November 2025 to February 2026. Inclusion criteria: (1) Full-time nursing students; (2) Clinical internship duration ≥ 8 months; (3) Provided informed consent and voluntarily participated in the study. Exclusion criteria: (1) Absence from clinical practice for ≥ 1 month due to illness, leave, or other reasons; (2) Presence of mental disorders or psychiatric diseases; (3) Early termination of internship for any reason. This study was a cross-sectional survey. The required sample size should be at least 5 to 10 times the number of independent variables (34). Considering a 10% attrition rate, a minimum of 750 samples was required.

### 2.2 Data collection

Before the investigation, all members of the research team received unified and standardized training to clarify the investigation process, questionnaire requirements, and quality control standards. Each researcher was responsible for the investigation in 1 to 2 hospitals, and questionnaire collection and quality control were completed with the help of a questionnaire platform. Prior to the investigation, communication was conducted with the nursing department of each hospital to obtain their consent and cooperation. Questionnaires were distributed through three methods: first, researchers directly sent the questionnaire QR code and filling instructions to the WeChat groups of pre-professional nursing students; second, electronic questionnaires were sent to hospital contacts, who distributed them conveniently according to the standards; third, during the post-internship forum, investigators distributed paper questionnaires on-site, invited interns to participate voluntarily, and attached unified instructions. The first page of the questionnaire explained the purpose, significance, methods, and filling requirements of the investigation. Participants filled in the questionnaire voluntarily and anonymously, and all items were mandatory. To ensure data accuracy, the questionnaire was set to prohibit repeated answers from the same IP address and restrict each WeChat account to only one submission. After questionnaire collection, two researchers conducted double-checks on the filling quality on the same day, verified and cleaned the data, and manually excluded invalid questionnaires. The criteria for invalid questionnaires were as follows: consistent answers or obvious patterns, contradictory logic or incorrect answers to trap questions, and response time < 160 s. A total of 750 questionnaires were collected in this investigation. Among them, 38 invalid questionnaires were excluded (33 due to regular answers, logical contradictions or abnormal information, and 5 due to completely consistent scale answers), resulting in 712 valid questionnaires with an effective recovery rate of 94.93%.

## 3 Measurements

### 3.1 General information questionnaire

A general information questionnaire was used to collect participants’ characteristics, including Gender, Place of residence, Parental Expectation Level, Attitude toward the Nursing Profession, Attitude toward Career Prospects, Holding a Position in Class/Club, Academic record, Understanding of basic AI algorithms, Has received AI-related training, AI tool usage frequency, Use of AI software in clinical practice/learning.

### 3.2 Generative artificial intelligence literacy scale for college students

This scale was developed by Liu et al. (35), consisting of 38 items, including 5 s-order dimensions: Awakening of GenAI Awareness, Knowledge Construction of GenAI, Development of GenAI competencies, Shaping of GenAI mindset, and Ethical Cognition of GenAI; and 13 third-order dimensions: Inquiry Awareness, Competence Awareness, Technological Content Knowledge, Technological Practical Knowledge, Problem Construction, Cognitive Interaction, Collaborative Regulation, Iterative Reflection, Computational Thinking, Critical Thinking, Interdisciplinary Innovative Thinking, Moral Ethics, and Safety Ethics. A 5-point Likert scale was adopted for the scale, where 1 point indicated “strongly disagree” and 5 points indicated “strongly agree.” The total score ranged from 38 to 190, with higher scores indicating higher levels of individual GenAI literacy. The internal consistency coefficients of each dimension ranged from 0.7 to 0.83. In this study, the overall Cronbach’s *α* coefficient was 0.90, and the coefficients of each dimension ranged from 0.68 to 0.75.

### 3.3 Nursing practice readiness scale

This scale was developed by Kim et al. (36) to assess the workforce readiness of newly graduated nurses in terms of knowledge, skills, and attitudes. Zhang et al. (37) adapted it into a Chinese version, which consists of 35 items divided into 5 dimensions: Clinical Judgment and Nursing Performance, Professional Attitude, Patient-Centeredness, Self-Regulation, and Collaborative Interpersonal Relationships. A 5-point Likert scale was used, with scores ranging from 1 to 5 points (from “strongly disagree” to “strongly agree”). The total score ranged from 35 to 140, with higher scores indicating a higher level of workforce readiness. This scale was applied to the population of pre-professional nursing students in this study, with an overall Cronbach’s *α* coefficient of 0.91 and dimension-specific coefficients ranging from 0.83 to 0.92. The CFA fitting indices were reported as follows:*χ^2^*/df = 1.975, RMSEA = 0.037, CFI = 0.954, TLI = 0.949, NFI = 0.912. All indicators reached the acceptable psychometric standard, which confirmed that the original factor structure of the NPRS is applicable and has good structural validity in the population of nursing interns.

### 3.4 Data analysis

LPA was performed using Mplus 8.3 software. The number of classes was gradually increased starting from 1 class until the model fit indices reached the optimal level. The model fit indices included: ①Akaike Information Criterion (AIC), Bayesian Information Criterion (BIC), and sample size-adjusted BIC (aBIC); smaller values indicated better model fit. ②Entropy, which ranged from 0 to 1.0; a value closer to 1.0 indicated more accurate classification, and an entropy value of approximately 0.8 suggested a classification accuracy of > 90%. ③Lo–Mendell–Rubin adjusted likelihood ratio test (LMR) and Bootstrapped likelihood ratio test (BLRT), which reflected the differences in fit among latent profile models; *p* value < 0.05 indicated that the model with k classes was superior to the model with k-1 classes (38).

Statistical analysis was conducted using SPSS 27.0 software. Measurement data conforming to normal distribution were expressed as mean ± standard deviation, while those not conforming to normal distribution were expressed as median and interquartile range. Missing data were replaced with the mean value. Count data were expressed as frequency and percentage. Chi-square test was used for inter-group comparison of unordered categorical variables, and Kruskal-Wallis H test was used for inter-group comparison of ordered categorical variables. Logistic regression analysis was employed to explore the influencing factors of different categories of GenAI literacy and workforce readiness among undergraduate pre-professional nursing students, with *p* < 0.05 considered statistically significant.

## 4 Results

### 4.1 General information

A total of 712 pre-professional nursing students were included, with a mean age of 21.50 ± 0.83 years. The demographic and baseline characteristics of the participants are presented in Table 1.

**Table 1:** A univariate analysis of potential types of GenAI literacy for undergraduate pre-professional nursing students.

Variables	Total	C1	C2	C2	*χ^2^/F*	*p*
	(*n* = 712) *n*(%)	(*n* = 201) *n*(%)	(*n* = 399) *n*(%)	(*n* = 112) *n*(%)		
Gender					4.244	0.120
Male	93(13.1)	27(13.4)	58(14.5)	8(7.1)		
Female	619(86.9)	174(86.6)	341(85.5)	104(92.9)		
Place of residence					4.384	0.357
Urban	108(15.2)	33(16.4)	58(14.5)	17(15.2)		
Town	172(24.2)	47(23.4)	90(22.6)	35(31.3)		
Rural	432(60.7)	121(60.2)	251(62.9)	60(53.6)		
Parental Expectation Level					2.312	0.679
Low	216(30.3)	69(34.3)	113(28.3)	34(30.4)		
Uncertain	285(40.0)	75(37.3)	165(41.4)	45(40.2)		
High	211(29.6)	57(28.4)	121(30.3)	33(29.5)		
Attitude toward the Nursing Profession						
Negative	6(0.8)	3(1.5)	3(0.8)	0(0)	0.407	0.816
Neutral	188(26.4)	52(25.9)	108(27.1)	28(25.0)		
Positive	518(72.8)	146(72.6)	288(72.2)	84(75.0)		
Attitude toward Career Prospects					6.583	0.160
Seldom	84(11.8)	17(8.5)	52(13.0)	15(13.4)		
Uncertain	496(69.7)	154(76.6)	267(66.9)	75(67.0)		
Positive	132(18.5)	30(14.9)	80(20.1)	22(19.6)		
Holding a Position in Class/Club					1.195	0.550
Yes	366(51.4)	107(53.2)	198(49.6)	61(54.5)		
No	346(48.6)	94(46.8)	201(50.4)	51(45.5)		
Academic record					40.745	<0.001
Very Poor	17(2.4)	3(1.5)	12(3.0)	2(1.8)		
Below Average	56(7.9)	15(7.5)	39(9.8)	2(1.8)		
Average	362(50.8)	136(67.7)	189(47.4)	37(33.0)		
Outstanding	277(38.9)	47(23.4)	159(39.8)	71(63.4)		
Understanding of basic AI algorithms					74.148	<0.001
No	503(70.6)	182(90.5)	270(67.7)	51(45.5)		
Yes	209(29.4)	19(9.5)	129(32.3)	61(54.5)		
Has received AI-related training					22.384	<0.001
No	389(54.6)	146(72.6)	225(56.4)	18(16.1)		
Yes	323(45.4)	55(27.4)	174(43.6)	94(83.9)		
AI tool usage frequency					149.892	<0.001
Almost never used	37(5.2)	21(10.4)	14(3.5)	2(1.8)		
1–3 days per week,	234(32.9)	104(51.7)	126(31.6)	4(3.6)		
4–6 days per week,	298(41.9)	65(32.3)	192(48.1)	41(36.6)		
Daily	143(20.0)	11(5.5)	67(16.8)	65(58.0)		
Use of AI software in clinical practice/learning					71.287	<0.001
No	262(36.8)	118(58.7)	129(32.3)	15(13.4)		
Yes	450(63.2)	83(41.3)	270(67.7)	97(86.6)		

C1, Low GenAI literacy group; C2, Moderate GenAI literacy group; C3, High GenAI literacy group.

### 4.2 Latent profiles of GenAI literacy among pre-professional nursing students

LPA was conducted with the five dimension scores of GenAI literacy as the observed indicators. The optimal number of profiles was determined by sequentially increasing the number of classes from 1 to 5. The fit indices of the five models are presented in Table 2. Detailed model comparisons were performed as follows:

**Table 2:** Fit indices of latent profile models for GenAI literacy among undergraduate pre-professional nursing students (*n* = 712).

Profile	K	AIC	BIC	aBIC	Entropy	LMR	BLRT	Proportion
1	10	19093.840	19139.521	19107.769	-	-	-	-
2	16	18082.467	18155.556	18104.752	0.823	<0.001	<0.001	0.692/0.308
3	22	17724.554	17825.051	17755.196	0.801	0.014	<0.001	0.282/0.560/0.158
4	28	17593.312	17721.218	17632.311	0.795	0.045	<0.001	0.180/0.060/0.232/0.528
5	34	17541.613	17696.928	17588.970	0.803	0.069	<0.001	0.017/0.480/0.249/0.049/0.205

When the number of classes was set to 5, the AIC, BIC, and aBIC values were the lowest (indicating the best absolute fit), but the LMR test was not significant (*p* > 0.05), suggesting that the 5-class model did not significantly improve model fit compared with the model with fewer classes. When 4 classes were extracted, the LMR test was significant (*p* < 0.05), but the entropy value was below 0.8, indicating relatively low classification accuracy. For the 2-class model, although it yielded the highest entropy value of 0.823 with ideal classification accuracy, its AIC, BIC, and aBIC values were higher than those of the 3-class model, reflecting a poorer overall model fit. When 3 classes were specified, the entropy value was 0.801, which was within an acceptable excellent range for classification; meanwhile, the AIC, BIC, and aBIC values were comparatively lower, and both the LMR and BLRT tests were statistically significant (*p* < 0.05). In addition, the proportion of each class was reasonable and interpretable in professional context. After comprehensive consideration of information criteria, entropy value, significance of LMR/BLRT, and practical interpretability, the 3-class model was ultimately selected as the optimal solution (see Table 2).

### 4.3 Naming of each latent category

**Figure 1:**
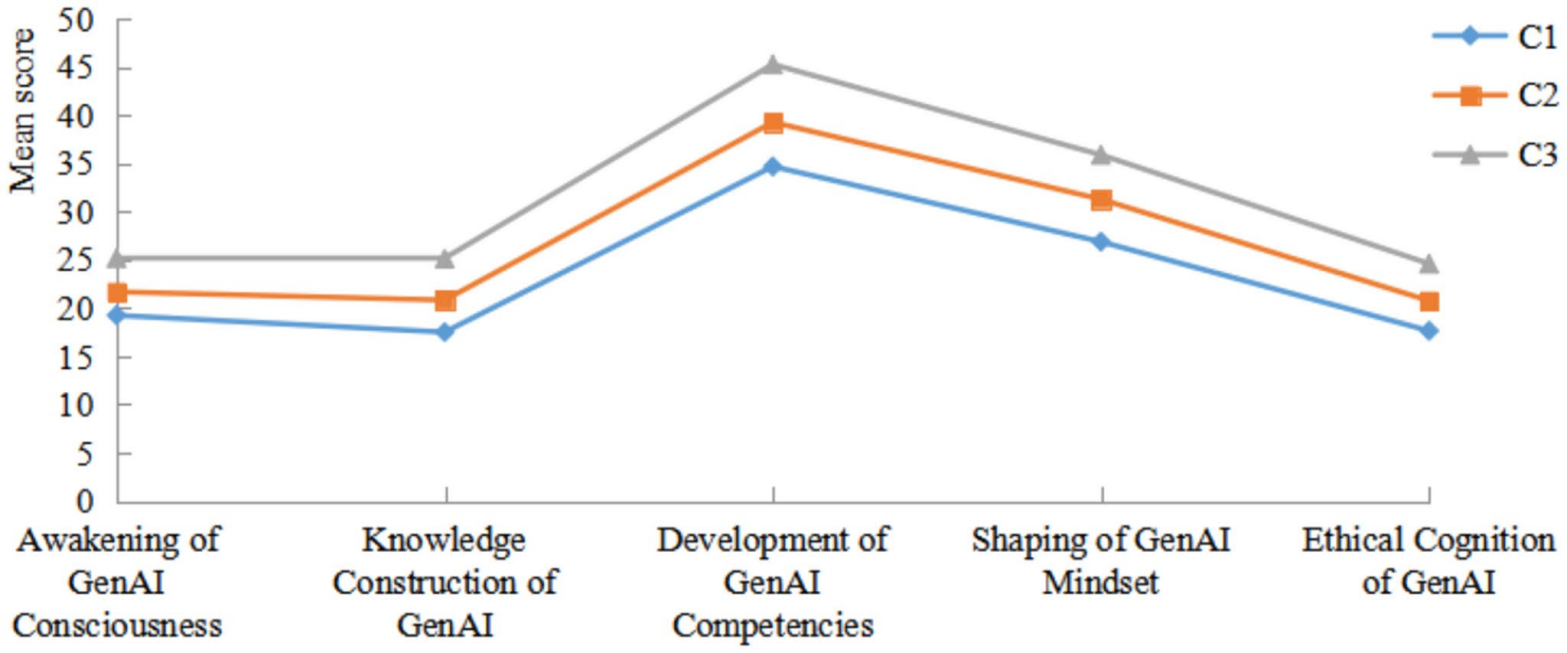
Distribution of GenAI literacy characteristics across three potential categories among undergraduate pre-professional nursing students.

According to the results of latent profile analysis, Figure 1 presents the mean scores of the five dimensions of GenAI literacy across the three latent classes among pre-professional nursing students. The results indicated statistically significant differences in the mean scores of all five dimensions among the three classes (*p* < 0.05), with a consistent overall trend across classes. Specifically, Class C1 comprised 201 pre-professional nursing students (28.2% of the total sample), whose scores on all five dimensions of GenAI literacy were lower than those of Class C2 and Class C3; thus, this class was labeled the low GenAI literacy group. Class C2 included 399 pre-professional nursing students (56.0% of the total sample) and was designated the moderate GenAI literacy group. Class C3 consisted of 112 pre-professional nursing students (15.8% of the total sample), whose scores on all five dimensions were higher than those of Class C1 and Class C2; accordingly, this class was named the high GenAI literacy group.

### 4.4 Univariate analysis of latent profiles of GenAI literacy

Chi-square test and Kruskal-Wallis H test were used to compare the distribution of single risk factors among pre-professional nursing students with different GenAI literacy latent profiles. The results are presented in Table 1.

### 4.5 Multinomial logistic regression of potential profiles of GenAI literacy

Multinomial logistic regression analysis was performed with the classification of GenAI literacy as the dependent variable and Class C1 as the reference group. Five variables with statistical significance in the univariate analysis were included as independent variables. For Class C2 compared with Class C1: Academic record [Average (OR = 0.619; 95% CI: 0.404–0.948)], Has received GenAI-related training (OR = 0.329; 95% CI: 0.191–0.568), GenAI tool usage frequency [Almost never used (OR = 0.210; 95% CI: 0.077–0.573); 1–3 days per week (OR = 0.339; 95% CI: 0.160–0.721)],and Use of GenAI software in clinical practice/learning (OR = 0.531; 95% CI: 0.361–0.781)were significant predictors. For Class C3 compared with Class C1: Academic record [Average (OR = 0.436; 95% CI: 0.237–0.803)], Has received GenAI-related training (OR = 0.269; 95% CI: 0.136–0.532), Understanding of basic GenAI algorithms (OR = 0.214; 95% CI: 0.109–0.423), GenAI tool usage frequency [Almost never used (OR = 0.109; 95% CI: 0.020–0.602); 1–3 days per week (OR = 0.027; 95% CI: 0.008–0.096); 4–6 days per week (OR = 0.194; 95% CI: 0.087–0.433)],and Use of GenAI software in clinical practice/learning (OR = 0.391; 95% CI: 0.192–0.796)were significant predictors. Detailed information is shown in Table 3.

**Table 3:** Multinomial logistic regression analysis of influencing factors of GenAI literacy (*n* = 712).

Variables	*B*	SE	*Wald χ^2^*	*p*	OR	95%CI
C2 VS C1						
Academic record(ref: Outstanding)						
Very Poor	0.841	0.697	1.458	0.227	2.32	0.592,9.089
Below Average	0.231	0.368	0.393	0.530	1.26	0.612,2.591
Average	−0.479	0.218	4.856	0.028	0.619	0.404,0.948
Understanding of basic GenAI algorithms(ref: Yes)	−0.13	0.217	0.358	0.550	0.878	0.574,1.343
Has received GenAI-related training(ref: Yes)	−1.111	0.278	15.947	<0.001	0.329	0.191,0.568
GenAI tool usage frequency(ref: Daily)						
Almost never used	−1.563	0.513	9.278	0.002	0.21	0.077,0.573
1–3 days per week	−1.08	0.384	7.915	0.005	0.339	0.16,0.721
4–6 days per week	−0.471	0.371	1.611	0.204	0.625	0.302,1.292
Use of GenAI software in clinical practice/learning(ref: Yes)	−0.633	0.197	10.36	0.001	0.531	0.361,0.781
C3 VS C1						
Academic record(ref: Outstanding)						
Very Poor	1.058	1.015	1.088	0.297	2.881	0.394,21.054
Below Average	−1.333	0.837	2.54	0.111	0.264	0.051,1.358
Average	−0.83	0.311	7.106	0.008	0.436	0.237,0.803
Has received GenAI-related training(ref: Yes)	−1.312	0.347	14.265	<0.001	0.269	0.136,0.532
Understanding of basic GenAI algorithms(ref: Yes)	−1.54	0.347	19.751	<0.001	0.214	0.109,0.423
GenAI tool usage frequency(ref: Daily)						
Almost never used	−2.216	0.872	6.458	0.011	0.109	0.02,0.602
1–3 days per week	−3.613	0.646	31.313	<0.001	0.027	0.008,0.096
4–6 days per week	−1.639	0.409	16.032	<0.001	0.194	0.087,0.433
Use of GenAI software in clinical practice/learning(ref: Yes)	−0.939	0.363	6.712	0.010	0.391	0.192,0.796

C1, Low GenAI literacy group; C2, Moderate GenAI literacy group; C3, High GenAI literacy group.

### 4.6 Professional workforce readiness with latent profile membership

Table 4 shows the differences among the three GenAI literacy groups on the five dimensions of the workforce readiness. For clinical judgment and nursing performance, professional attitudes, patient-centeredness, self-regulation, and collaborative interpersonal relationship among pre-professional nursing students, scores in the C3 group were significantly higher than those in the C1 and C2 groups, and scores in the C2 group were significantly higher than those in the C1 group, with statistically significant differences (*p* < 0.05).

**Table 4:** Professional GenAI literacy difference of two profiles (*n* = 712).

Variable	C1 (*n* = 201)	C*2* (n = 399)	C3(*n* = 112)	*F*	*p*
	*M ± SD*	*M ± SD*	*M ± SD*		
Clinical judgment and nursing performance	33.68 ± 3.77	39.71 ± 6.79	50.32 ± 8.92	235.297	<0.001
Professional attitudes	12.35 ± 3.09	14.61 ± 3.76	15.82 ± 4.66	37.229	<0.001
Patient-centeredness	12.06 ± 2.52	14.10 ± 2.94	17.23 ± 2.95	120.117	<0.001
Self-regulation	7.53 ± 2.81	8.52 ± 2.71	9.89 ± 2.47	27.689	<0.001
Collaborative interpersonal relationship	9.49 ± 2.09	11.21 ± 2.40	13.52 ± 2.32	111.536	<0.001

C1, Low GenAI literacy group; C2, Moderate GenAI literacy group; C3, High GenAI literacy group.

## 5 Discussion

The analysis of GenAI literacy among pre-professional nursing students reveals three distinct latent profiles: low, moderate, and high GenAI literacy, which collectively highlight the heterogeneity of AI competence in this critical population. The low GenAI literacy group constitutes 28.2% (*n* = 201) of the total sample. This significant proportion indicates that nearly one-third of nursing students exhibit notable deficiencies in GenAI literacy. These deficits often span core domains, including foundational GenAI knowledge, basic application skills, and ethical judgment, suggesting they lack the fundamental competencies required for roles integrated with AI technologies (39). Nursing students should approach Generative Artificial Intelligence tools rationally and apply them appropriately (40). Without upskilling, these students are not adequately prepared for professional contexts where AI workflows are increasingly prevalent. Educational strategies for this group should prioritize solidifying basic generative AI operational capabilities and fostering an understanding of risk identification (41). This includes training on ethical content generation, recognizing and mitigating potential algorithmic biases, and discerning misinformation (42). Given that low health literacy is a direct risk factor for patient safety, improving GenAI literacy here is crucial for preventing errors in AI-assisted healthcare environments.

The moderate GenAI literacy group represents the largest subgroup, accounting for 56.0% (*n* = 399) of the total sample. This finding suggests that the overall GenAI literacy level among the participants is moderate to high, with most students possessing basic GenAI knowledge and application skills sufficient for everyday scenarios (42). While this group can perform entry-level tasks, their readiness for advanced workplace applications of GenAI is conditional and necessitates structured onboarding and continuous support. Their competency aligns with a functional understanding of literacy, enabling basic application but often lacking the critical thinking and analytical skills required for in-depth interpretation and rational evaluation. This group also demonstrated the smallest standard deviation, indicating a highly homogeneous GenAI literacy level and minimal individual differences, which underscores its stability as the predominant population (41). Training for these students should focus on enhancing critical evaluation capabilities and iterative optimization (43). Key areas include advanced prompt engineering, multi-turn interactions for refining GenAI outputs, and strategies for seamlessly integrating GenAI tools into learning and research processes (44).

The high GenAI literacy group comprises 15.8% (*n* = 112) of the sample. Participants in this group demonstrate outstanding proficiency across GenAI knowledge, application, and ethical judgment, enabling profound and flexible utilization of GenAI technologies (42). These individuals are well-suited for complex, autonomous, and leadership roles that require advanced AI literacy, such as optimizing work efficiency and driving innovation within the healthcare sector (45). This advanced level of literacy is closely associated with critical digital literacy and algorithmic reflexivity, allowing them to analyze information from contextual and algorithmic bias perspectives, a core competency in the AI era. For students in this high-literacy subgroup, greater attention should be paid to the normative application of Generative AI, particularly regarding medical ethics and academic integrity, so as to standardize their rational and ethical use of GenAI in clinical practice, academic research, and professional learning (46).

This study identified academic performance, receipt of GenAI-related training, understanding of basic GenAI algorithms, GenAI tool usage frequency, and clinical GenAI application as the principal determinants of GenAI literacy classification. In terms of academic performance, there is typically a statistically significant difference between the ‘Outstanding’ group and the ‘Average’ group. High-achieving students demonstrated notable advantages in managing the cognitive load associated with GenAI, attributable to their superior information processing, concept integration, and critical thinking abilities (43). Cognitive Load Theory (CLT) further explains this finding: academically stronger students typically possess greater working memory capacity, more efficient automaticity, and better metacognitive monitoring (47), enabling them to adapt rapidly to GenAI complexity and leverage it more effectively for learning and innovation (48). However, the statistically non-significant differences observed between the ‘Outstanding’ group and the ‘Very poor”/"Below-averag’ groups may reflect pervasive cognitive resource depletion among low-achieving students, low variability in their GenAI usage behaviors, and potential limitations in the sensitivity of current measurement tools (49). Future research may require more fine-grained measurement approaches and targeted intervention strategies to better understand and support students with varying cognitive capacities in GenAI-augmented learning environments (50).

Conversely, students lacking algorithmic understanding and systematic training exhibited insufficient GenAI literacy, characterized by limited cognition of GenAI concepts, healthcare applications, and auxiliary value. Consequently, they struggled to recognize GenAI’s potential in nursing assessment, health guidance, and workflow optimization, and failed to develop informed trust in the technology (51). Without professional guidance, these students relied on informal channels for GenAI information, rendering them susceptible to misconceptions such as “GenAI will replace nurses,” which reinforced stereotypes and cognitive biases that further constrained literacy development (52). According to the Technology Acceptance Model (TAM), students with low GenAI literacy may underestimate its practical value due to unfamiliarity with functional boundaries, while perceived operational difficulty reduces their perceived ease of use (53). These findings carry critical implications for curriculum design, particularly pre-employment interventions. By aligning curricula with the identified influencing factors, universities can proactively cultivate GenAI literacy before workforce entry—leveraging high-achieving students’ cognitive strengths while implementing standardized training to narrow overall literacy gaps (54). This approach enhances adaptability to GenAI-integrated work environments and ensures future professionals possess robust GenAI literacy to meet the demands of increasingly AI-reliant clinical settings (55).

Infrequent use and limited application hinder GenAI literacy development, manifested as inadequate knowledge reserves and weak practical skills (52). Students consequently struggle to master GenAI application scenarios, operational protocols, and core functions, impairing their adaptability to GenAI-enhanced nursing practice (32). This diminishes self-efficacy and creates a vicious cycle between low literacy and poor application confidence (52)—further weakening motivation to learn, solidifying cognitive biases, and preventing the formation of a systematic GenAI literacy framework (56).

Students who rarely engage with GenAI tools in clinical settings cannot accumulate practical experience or intuitively appreciate GenAI’s enabling value in improving efficiency, optimizing decisions, and supporting health assessments (10). Moreover, insufficient literacy may lead to misunderstanding the collaborative relationship between GenAI and humanistic care, generating concerns that inappropriate GenAI application could undermine nursing’s humanistic attributes and threaten professional identity (51). To address these challenges, nursing education should systematically integrate GenAI into curricula through practice-oriented activities, critical thinking training, ethics education, and faculty development (10).

This study revealed that workforce readiness scores exhibited a significant gradient pattern—high-level > moderate-level > low-level GenAI literacy—demonstrating a clear positive association between GenAI literacy and workforce readiness. This provides direct empirical support for integrating GenAI literacy training into nursing education. The underlying mechanism is that high-literacy students can proficiently employ GenAI tools to supplement theoretical knowledge, simulate clinical scenarios, and optimize communication workflows, effectively bridging the classroom-practice gap and translating these advantages into stronger employability (52). Furthermore, GenAI can act as a virtual preceptor to support nursing students in simulated clinical reasoning and clinical decision-making training (57). It enables learners to repeatedly simulate clinical scenarios, refine diagnostic and care thinking, and compensate for the limitations of limited clinical internship resources, thereby further facilitating the transition from classroom theoretical knowledge to authentic clinical practice (57). In contrast, low-literacy students lack proficient operation and rigorous information evaluation capabilities, preventing effective use of GenAI to compensate for limited practical exposure. Moderate-literacy students possess basic operational skills but lack flexibility in integrating GenAI into practice. These cross-level disparities collectively account for the observed gradient in clinical performance and workforce readiness, consistent with evidence on GenAI’s value in enhancing nursing education efficiency and decision-making (58). In practice, nursing colleges should develop tiered training programs, clinical institutions should establish GenAI practice platforms, and the profession should strengthen ethical standards. Simultaneously, students should be guided to proactively enhance their GenAI literacy, facilitating deep integration of GenAI with nursing practice and ultimately preparing nursing professionals for the era of intelligent healthcare.

### 5.1 Limitations

This study still has several limitations. First, convenience sampling was adopted, so there may be certain limitations in the representativeness of the sample and the generalizability of the research results. Future studies need to provide diverse stratified samples. Second, we used the NPRS to evaluate the workforce readiness of pre-professional nursing students, but this scale was developed for newly recruited nurses and needs further validation in the nursing intern population. In addition, the Cronbach’s *α* coefficients of several subdimensions of the Generative Artificial Intelligence Literacy Scale ranged from 0.68 to 0.75, with individual dimensions slightly lower than the widely recognized cutoff value of 0.70. Marginal internal consistency may bring potential reliability concerns to the latent profile analysis results. Therefore, the relevant grouping findings should be interpreted cautiously. Future studies could appropriately revise and screen scale items to further improve the internal consistency of subscales. Third, as a cross-sectional study, the results of this study cannot be used to determine a causal relationship. More longitudinal studies are needed in the future to track the trajectory of changes in pre-professional nursing students’ GenAI literacy over time and clarify the mechanism of action between the two. Final, constructing a scatter plot is of great value for exploring the continuous association between GenAI literacy and Workforce Readiness, as well as testing potential linear or nonlinear (e.g., U-shaped) relationships. It is recommended that future studies further adopt scatter plot methods to verify linear or nonlinear associations.

## 6 Conclusion

This study employed LPA to systematically identify the subgroup characteristics and predictive factors of GenAI literacy among pre-professional nursing students, providing empirical support for nursing talent cultivation and clinical practice optimization. The results indicated that GenAI literacy among pre-professional nursing students presents three distinct latent profiles: low-level, moderate-level, and high-level GenAI literacy groups. This study clearly identified the key predictive factors influencing the subgroup classification of pre-professional nursing students’ GenAI literacy, including academic performance, understanding of GenAI algorithms, receipt of GenAI-related special training, daily usage frequency of GenAI tools, and practical application of GenAI software in clinical practice and professional learning. Furthermore, Higher GenAI literacy is positively associated with greater nursing practice readiness among nursing interns, which is crucial for their smooth transition to clinical nursing work and the improvement of care quality. Based on the heterogeneity of GenAI literacy among pre-professional nursing students, this study suggests that nursing managers design targeted intervention measures and special training programs in future practice. In summary, enhancing GenAI literacy is essential for pre-professional nursing students to meet clinical needs, as it directly improves their workforce readiness and equips them to adapt to the intelligent healthcare era.

## Data Availability

The original contributions presented in the study are included in the article/supplementary material, further inquiries can be directed to the corresponding author.
